# The C-terminal structure of the N^6^-methyladenosine deaminase YerA and its role in deamination

**DOI:** 10.1042/BCJ20240728

**Published:** 2025-02-12

**Authors:** Qian Jia, Hui Zeng, Nan Xiao, Jing Tang, Shangfang Gao, Wei Xie

**Affiliations:** 1 MOE Key Laboratory of Gene Function and Regulation, State Key Laboratory for Biocontrol, School of Life Sciences, Sun Yat-Sen University, Guangzhou, Guangdong 510006, People’s Republic of China

**Keywords:** catalytic mechanism, deaminase, molecular docking, N^6^-methyladenosine, substrate specificity

## Abstract

The N^6^-methyladenine (6mA) modification is an essential epigenetic marker and plays a crucial role in processes, such as DNA repair, replication, and gene expression regulation. YerA from *Bacillus subtilis* is considered a novel class of enzymes that are capable of catalyzing the deamination of 6mA to produce hypoxanthine. Despite the significance of this type of enzymes in bacterial self-defense system and potential applications as a gene-editing tool, the substrate specificity, catalytic mechanism, and physiological function of YerA are currently unclear due to the lack of structural information. In the present study, we expressed the recombinant enzyme and conducted its reconstitution to yield the active form. Our deamination assays showed that N^6^-methyladenosine (N^6^-mAdo) served as a more favorable substrate than its base derivative 6mA. Here, we report the high-resolution structure of the C-terminal region of YerA, which exhibited a compact architecture composed of two antiparallel β-sheets with no obvious close structural homologs in Protein Data Bank. We also created docking models to investigate the ligand-binding pattern and found that more favorable contacts of N^6^-mAdo with the enzyme-binding pocket lead to its preference for N^6^-mAdo over 6mA. Finally, structural comparison of the N^6^-methyladenosine monophosphate deaminase allowed us to propose that a plausible role for this C-terminal region is to shield the active site from solvent and protect the intermediate during catalysis. Taken together, this study sheds light on the catalytic mechanism and evolutionary pathways of the promiscuous enzyme YerA, thereby contributing to our molecular understanding of epigenetic nucleoside metabolism.

## Introduction

Methylation is a significant epigenetic modification in nucleic acids. In DNA, 5-methylcytosine is the most prominent modification in mammals, whereas N^6^-methyladenine (6mA) is most common in prokaryotes [1]. This epigenetic modification plays a pivotal role in processes such as DNA repair, replication, and gene expression regulation, among others, and also aids in protecting organisms from the invasion of foreign species [2,3]. 6mA primarily fulfills its function through the restriction–modification (R–M) system. In bacteria, the R–M system comprises restriction endonucleases and methyltransferases, which cooperate to selectively recognize and subsequently degrade invading species with foreign DNA [4]. On the other hand, 6mA present in RNA (referred to as the m^6^A modification) is an active research topic in life sciences in recent years. This modification was first discovered by Desrosiers et al. in the 1970s and was later found to be ubiquitous on mRNA [5]. In 2011, it was established that the m^6^A modification is reversible, with the fat mass and obesity-associated protein acting as a demethylase (dubbed the ‘eraser’) to remove the methyl group from m^6^A [6]. Since then, research on this topic has grown rapidly, revealing diverse functions of m^6^A in disease regulation and tumorigenesis [7,8].

Although 6mA is the most abundant DNA epigenetic modification in prokaryotes, it was not until 2015 that 6mA was first discovered in the genetic material of eukaryotes, such as *Drosophila* and *Caenorhabditis elegans* [2,9,10]. The presence of 6mA in the genomes of mammals and higher plants implies that it may play a role in their growth, development, and disease regulation, among other processes [11–13]. Additionally, 6mA is also found in higher concentrations in mitochondrial genomes [14].

**Figure 1:**
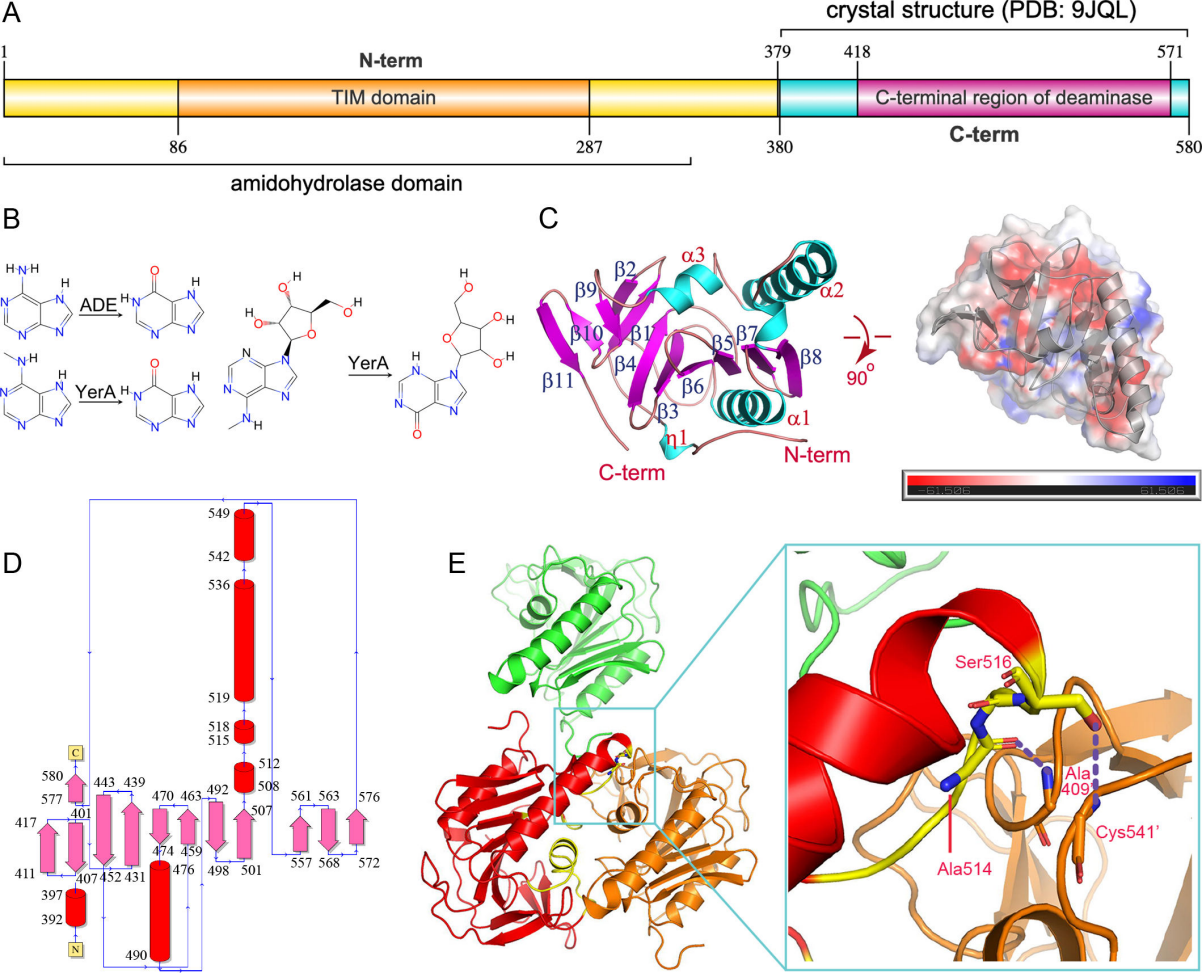
The chemistry and overall structure of YerA. (**A**) Schematic showing the domains of the full -length YerA. (**B**) The chemical reactions catalyzed by ADE and YerA, respectively. (**C**) The overall structure of YerA^Gly380-Arg580^, with helices colored cyan and strands colored magenta. Two orthogonal views are shown, and one view displays the surface charge distribution of YerA. The major secondary elements, including the N- and C -termini, are labeled. (**D**) The topology organization of YerA. (**E**) The crystal packing areas that form large intermolecular contacts. The intermolecular hydrogen bonds are indicated by the red dashed lines.

Purines, primarily in the form of purine nucleosides such as adenosine or guanosine, serve as the fundamental building blocks for the synthesis of ribonucleic acid (RNA) and deoxyribonucleic acid (DNA) and play pivotal roles in energy supply and metabolic regulation. Adenine deaminase (ADE) catalyzes the deamination of adenine to produce hypoxanthine (Figure 1A). Hypoxanthine is subsequently oxidized to uric acid by xanthine oxidase via the xanthine intermediate. This reaction is crucial in purine catabolism and the nucleotide salvage pathway [15]. In humans, purine metabolism is intricately linked to cellular physiological activities and has profound implications for health. Disruptions in purine metabolism can lead to severe diseases [16]. ADE belongs to the amidohydrolase superfamily (AHS). Enzymes in this family are also capable of catalyzing the deamination of adenosine, guanine, cytosine, and S-adenosylhomocysteine [17]. The AHS deaminases often exhibit a distorted (β/α)_8_ barrel structure, and their active sites include binding sites for divalent metal ions. In 2011, Kamat et al. reported the crystal structure of the ADE from *Agrobacterium tumefaciens* (Atu4426) in a Mn^2+^-bound form at a 2.2 Å resolution (PDB 3NQB) and proposed the catalytic mechanism for this enzyme [18,19]. They also deposited the structures of iron (II)-bound forms of Atu4426 (PDBs 3T81 and 3T8L) and studied its inactivation mechanism by H_2_O_2_ [20]. Overall, these metal ions typically bind to histidine residues at the end of β-sheets of the barrels, activating water molecules to initiate nucleophilic attacks.

The *yerA* gene from *Bacillus subtilis* encodes the protein YerA (Bsu06560) (Figure 1A). This protein shares 71% similarity and 52% identity with its homologous counterpart, Bh0637, from *Bacillus halodurans*. Through computational simulations, Siddhesh et al. found that Bh0637 has a similar active site to that of Atu4426 [19]. Their *in vitro* experiments demonstrated that both Bh0637 and YerA specifically recognize and catalyze the deamination of 6mA to hypoxanthine (Figure 1B), while showing no catalytic effects on unmodified adenine [19]. These findings suggested that YerA may play a critical role in epigenetic modifications. Additionally, YerA has been shown to possess conserved key amino acid residues that form a binuclear metal center [20]. Although YerA exhibits *in vitro* catalytic activity in the presence of Fe²^+^, Mn²^+^, and Zn²^+^ ions, the nature of its physiological metals remains unclear [19]. About 10 years later, Jiang et al. discovered that Bsu06560 is capable of deaminating N^6^-methyladenosine (N^6^-mAdo) and probably plays a role in the regulation of bacterial metabolism. They also created a substrate-binding model and performed mutagenesis studies to verify the contribution of key residues [21].

YerA is the first identified binuclear metal-dependent enzyme that specifically catalyzes the hydrolytic deamination of 6mA, representing a novel class of deaminases. However, the structural basis for its substrate recognition and catalytic mechanism remains unclear, particularly as the identity of its genuine substrate continues to be debated. Previously, our laboratory has been responsible for the structure determination of relevant deaminases and has conducted numerous mechanistic studies and functional characterization on these enzymes [22–27]. In particular, we conducted biochemical and structural studies on N^6^-methyladenosine monophosphate (N^6^-mAMP) deaminase (MAPDA), which also contains a (β/α)_8_ triose-phosphate isomerase (TIM)-barrel domain and a typical zinc-binding site [22]. MAPDA displays deamination activity toward N^6^-mAMP but not adenosine or AMP. Compared with YerA, MAPDA is a much smaller protein, approximately half the size of YerA. In this study, we conducted crystallographic, biochemical, and docking studies on YerA, which provide insights into the biochemical features and evolutionary pathways of this intriguing enzyme.

## Results

### Structure overview and comparison of YerA homologs

The full-length YerA protein exhibited robust expression in *Escherichia coli* but failed to produce crystals. To obtain diffracting YerA crystals, we systematically truncated regions from both termini, creating constructs including Ala15-Gly182, Ala15-Asp327, Ala15-Pro576, Ala15-Arg580, Ala15-Pro384, Pro26-Gln325, Pro26-Ala371, Pro26-Gly380, Pro26-Asp390, Pro26-Arg580, Gly76-Gly306, Gly76-Gly366, Gly76-Arg580, Pro81-Ser456, Gly82-Ala285, Pro90-Asp328, Asn95-Thr373, Asn95-Gly380, Thr181-Arg580, Ala235-Arg580, Ser286-Arg580, Gln325-R580, Gly366-Arg580, and Gly380-Arg580 (Supplementary Table S1). Proteins from most constructs were refractory to the expression in *E. coli*. Despite extensive trials, only two constructs pET-28a(+)/YerA^Ala15-Arg580^ and pET-28a(+)/YerA^Gly380-Arg580^ successfully produced crystals, after removing the N-terminal 6 × His affinity tag. While the former construct produced poor-diffracting crystals, pET-28a/YerA^Gly380-Arg580^ readily crystallized with a cubic morphology in ammonium sulfate or potassium phosphate as the precipitants. Optimal crystal growth conditions were 1.5 M KH_2_PO_4_ and 0.1 M HEPES pH 7.0, yielding high-quality crystals with excellent diffraction resolutions.

The cubic crystals belonged to a tetragonal system, with the space group P4_1_2_1_2, and each asymmetric unit contains one molecule with an estimated solvent content of 51%. Continuous and well-defined electron density was observed for the entire polypeptide chain, except for the first two residues introduced by cloning. The refined model comprised 201 amino acids and 101 water molecules, with refinement statistics summarized in Table 1.

**Table 1:** Data collection and refinement statistics.

PDB code	9JQL
**Data collection**	
Space group	*P*4_1_2_1_2
Cell dimensions	
*a*, *b*, *c* (Å)	103.17, 103.17, 42.63
a, b, g (°)	90, 90, 90
Resolution (Å)	23.76–2.10 (2.21–2.10)^1^
*R*_merge_	0.118 (0.682)
*I* / σ*I*	18.2 (3.9)
Completeness (%)	99.9 (100.0)
Redundancy	10.4 (10.8)
**Refinement**	
Resolution (Å)	23.76–2.10 (2.26–2.10)
No. reflections	13926
*R*_work_ / *R*_free_ (%)	22.60 / 27.06
No. atoms	
Protein	1573
Water	101
*B*-factors	
Protein	28.82
Water	33.36
Root-mean-square deviations	
Bond lengths (Å)	0.003
Bond angles (°)	0.608
Ramachandran favored (%)	95.48
Allowed (%)	4.52
Outliers (%)	0.00

1 Values in parentheses are for highest-resolution shell.

The YerA^Gly380-Arg580^ structure adopts an α+β fold with a globular shape, divisible into two domains: an N-terminal β-sheet of eight antiparallel strands flanked by helices (Gly380-Leu550) and a C-terminal β-sheet featuring three antiparallel strands (Gln551-Arg580) (Figure 1C,D and Supplementary Figure S1). These domains are spatially close, forming a compact structure devoid of central cavities or concave surfaces. The electrostatic surface exhibits an even charge distribution, with a theoretical isoelectric point of approximately 5.0.

Analysis by the PISA server indicated that YerA^Gly380-Arg580^ does not form an oligomeric structure within the crystal lattice. The calculated total buried surface area is 958.9 Å², with the largest intermolecular contacts involving Ser462-Gly468, Gly512-Ser516, and Glu539-Leu555. However, the second region forms crystal contacts through backbone hydrogen bonds between Ala514 and Ala409’ (of a symmetry mate), and Ser516 and Cys541’ (Figure 1E). The monomeric state is also consistent with that of the full-length protein as indicated by the size-exclusion chromatography (data not shown).

### Sequence and structure alignment with bacterial orthologs

**Figure 2:**
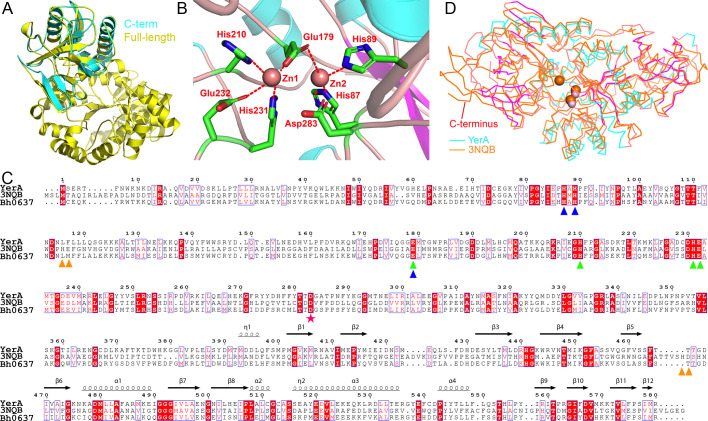
Structure and sequence comparison with orthologs from other organisms. (**A**) Structure overlay of YerA^Gly380-Arg580^ (magenta and cyan, PDB 9JQL) with the predicted AlphaFold full-length structure of YerA (cyan). The coloring scheme of YerA^Gly380-Arg580^ is identical toto that in Figure 1, and the TIM barrel of the full-length YerA is colored yellow. (**B**) The coordination pattern of the two zinc metal ions (Zn1 and Zn2) in YerA/AF-FL-2Zn. The metal ions are shown as pink spheres, and the ligation pattern is indicated by red dashed lines.(**C**) Sequence alignment of YerA with Atu4426 and Bh0637. The secondary structure of YerA is shown at the top. Identical residues in the sequences are shaded red, and similar residues are also highlighted in red. The ligands for Zn1, Zn2, and Zn3 are represented by green, blue, and orange triangles, respectively. The general base, Asp283, is marked with a red asterisk. (**D**) Structure overlay of YerA/AF-FL-2Zn (secondary structure colored as follows: cyan for helices, magenta for sheet, pink for loops, PDB 9JQL) with Atu4426 (orange, PDB 3NQB), represented by Cα traces.

We then compared the structure of YerA^Gly380-Arg580^ with that of the predicted full-length YerA protein using AlphaFold 3. The two proteins align with a root-mean-square deviation (RMSD) of 0.73 Å over 187 Cα atoms (Figure 2A). Significant structural discrepancies are observed at the N-, C-termini, and Pro507-Tyr519 regions. As mentioned, the latter region participates in crystal contacts, explaining the structural differences due to crystallization artifacts. The predicted structure revealed that the Pro86-Pro287 region forms the core catalytic structure of the TIM domain, while the C-terminal region is juxtaposed to the core and closely associated with the catalytic domain. The N-terminal fragment (Met1-Lys379) lacks clear boundaries within itself, explaining the poor heterologous expression levels observed during our extensive trials with truncated forms. The current 3D structure aligns with bioinformatics analysis, which indicated that the Met1-Thr360 region harbors the amidohydrolase domain (Pfam accession number PF01979), and Glu418-Lys571 represents the C-terminal region of deaminases (Pfam accession number PF13382) [28]. Inductively coupled plasma mass spectrometry result suggested that the recombinant enzyme contained Fe^2+^ or Zn^2+^ ions rather than Mn^2+^ (data not shown). AlphaFold 3 was used to place the two zinc metal ions and study their coordination patterns. As predicted, the metals are coordinated by His210, Glu179, His231, His87, His89, and Asp283, with Glu179 coordinating both metal ions simultaneously. Notably, three additional water molecules may participate in metal ligation (one for each metal ion and one being shared) but were not predicted by the algorithm (Figure 2B). This coordination pattern suggests a two-metal catalytic mechanism. When a third zinc metal ion was suggested, AlphaFold 3 failed to generate a plausible model, either with or without an AMP molecule (data not shown). Here, AMP is an optional ligand accepted by AlphaFold 3 and was used as an analog for N^6^-mAMP.

A DALI search for YerA^Gly380-Arg580^ homologs retrieved only remotely related structures with RMSD scores generally ranging from ~5 Å or above, with 70–90 aligned Cαs, indicating that the domain’s structure is unique. We compared the predicted full-length YerA structure with two zinc metals (named YerA/AF-FL-2Zn) to Atu4426 bound by Mn²^+^ ions (PDB 3NQB) or Fe³^+^ (PDB 3T81) (Figure 2C). These structures resemble YerA, with Atu4426 sharing 24% sequence identity and 39% sequence similarity with YerA across their entire sequences (Figure 2C,D). The proteins align with an RMSD value of 2.4 Å over 516 aligned Cα atoms. Conserved residues primarily cluster at the metal-binding site and the putative base-binding pocket.

According to the homologous structure PDB 3NQB, Atu4426 has three metal-binding sites and exhibits distinct substrate specificity by deaminating unmethylated adenine using two of the metal ions. Its catalytic metal-binding residues and metal ions roughly align with their counterparts in YerA/AF-FL-2Zn, with a translation of ~1.6 Å (Figure 2D). However, the residues responsible for coordinating the third metal ion are not conserved (Figure 2C), suggesting that YerA only utilizes two metals for catalysis. The Asp290 equivalent in Atu4426, Asp283 in YerA, acts as the general base to abstract the proton from the critical water nucleophile. Additionally, the remaining parts of the two structures show relatively large local variations, particularly at the C-terminus, indicating their distinct functional differences (Figure 2D).

### Substrate-recognition specificity

**Figure 3:**
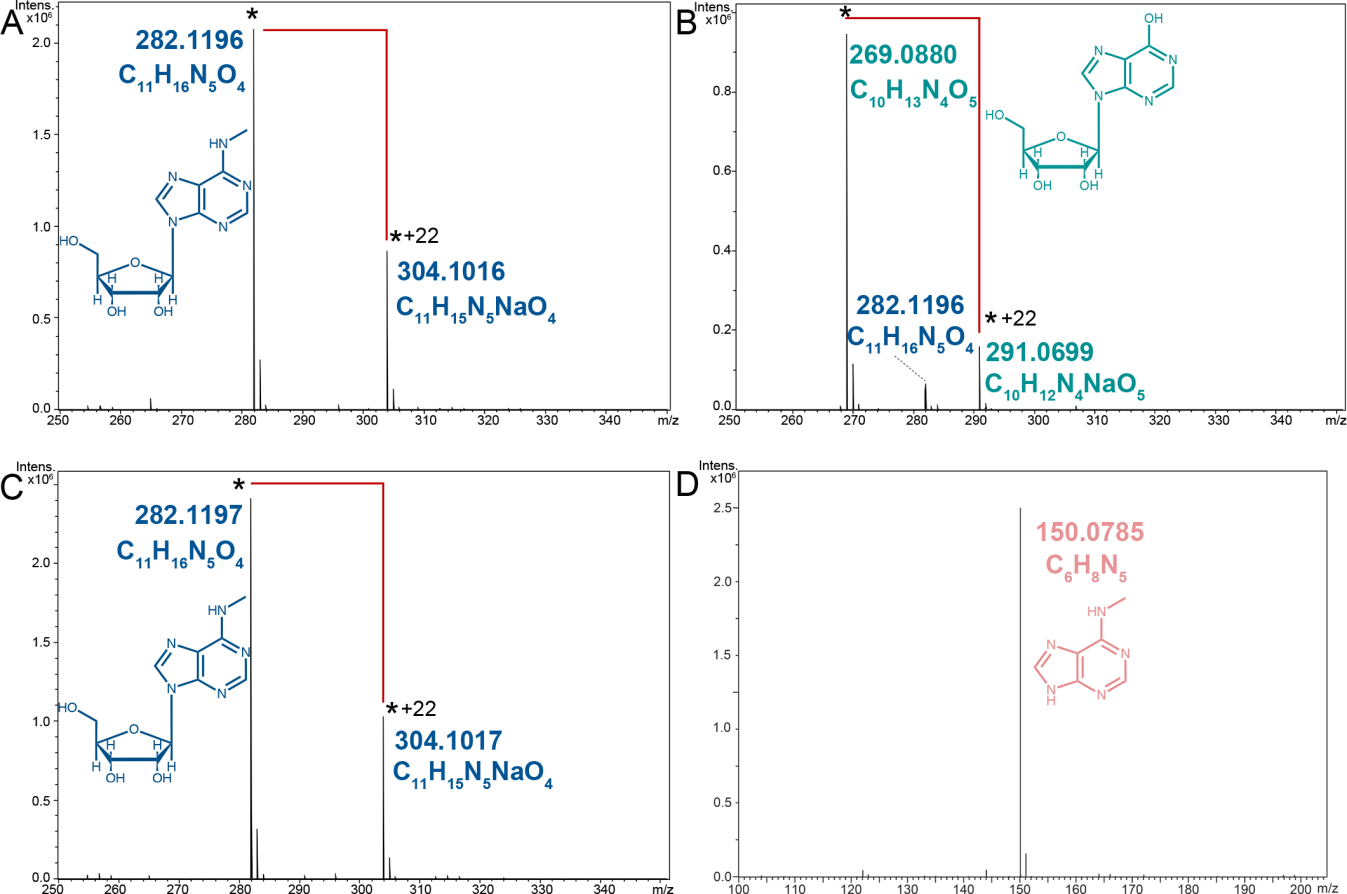
The deamination activity tests of YerA toward different substrates, analyzed by the LC-Q-TOF-MS. (**A**) The mass- spectrometry result of the N^6^-mAdo standard. (**B-C**) The 5-h deamination product of N^6^-mAdo to Ino by YerA after the reconstitution protocol (**B**); and without the reconstitution protocol (**C**). (**D**) The 5-h deamination product of 6mA by YerA. The peak for the molecular ions was indicated by the * signs, while the peak corresponding to the Na^+^-absorbed species was indicated by the ‘* + 22’ signs, and the set of the peaks were connected by the red solid lines. Abbreviation LC-Q-TOF,-MS, liquid chromatography-quadrupole time-of-flight mass spectrometry.

To confirm the substrate specificities and reconstitution efficiencies of YerA, deamination reactions involving N^6^mAdo/6mA were monitored using electrospray ionization mass spectrometry in positive ion mode. The molecular formula of inosine (Ino) is C_10_H_13_N_4_O_5_, and its molecular weight is 269.0880 g/mol, while the molecular formula of N^6^-mAdo is C_11_H_16_N_5_O_4_, with the molecular weight of 282.1196 g/mol. Figure 3A showed that the N^6^-mAdo standard showed a peak at 282.1196 g/mol and 304.1016 g/mol, respectively, indicating that the latter was a Na^+^ adduct. Following the metal reconstitution protocol, YerA deaminated the favored substrate N^6^-mAdo to Ino after a 5-h reaction, only a small amount of substrate remained, thereby validating the effectiveness of our holoenzyme reconstitution protocol (Figure 3B). Conversely, the negative control protein, YerA without the reconstitution process, failed to generate such a product, and the composition of the substrate did not change over the time course (Figure 3C). Notably, in our experiment using 6mA (C_6_H_8_N_5_, 150.0785 g/mol) and the same batch enzyme, we did not observe the corresponding product hypoxanthine (C_5_H_3_N_4_O, 135.0303 g/mol) within our reaction time frame (Figure 3D). Previous reports have indicated that YerA exhibits lower activity toward the base form of the methylated derivative compared with the nucleoside form. Consistent with the findings of Jiang et al., the catalytic efficiency for the nucleoside form is 28 times higher than that for the base form. Further *in vitro* functional investigations are required in this regard [21].

**Figure 4:**
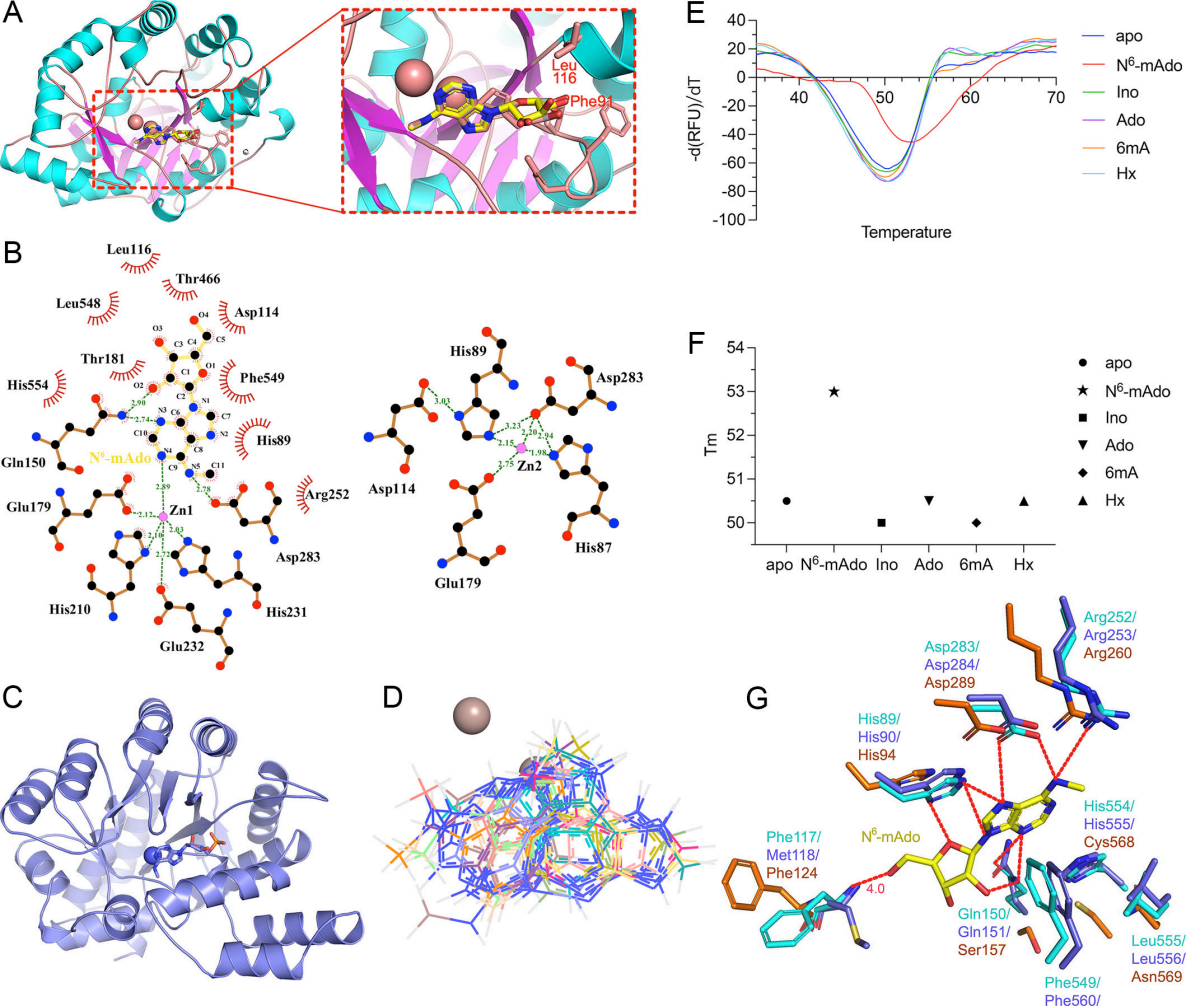
The docking models of YerA with different ligands. (**A**) The docking model of YerA with N^6^-mAdo. The two residues responsible for possible phosphate clashes (Leu116 and Phe93) are shown in sticks models. Only the TIM-barrel domain is shown for clarity. (**B**) The interaction pattern rendered by LigPlus, with hydrogen bonds indicated by the green dashed lines with the cutoff distance of 3.3 Å. (**C**) The binding pattern of N^6^-mAdo to the TIM-barrel domain of MAPDA (PDB 6IJM). The orientation of MAPDA is presented in the same view angle as YerA in (**A**). (**D**) Diverse potential conformations of the ligand 6mA bound at the active site of YerA. The two metals are shown as spheres, while the ligand in different orientations is shown as lines. (**E**) The melting curves of apo-YerA (without the reconstitution) in complex with various ligands. The horizontal axis indicates temperature, while the vertical axis indicates dRFU (relative fluorescence units)/dT. (**F**) The T_m_ values between the complexes and the apo-enzyme. apo:, apo-YerA; N^6^-mAdo:, N^6^-methyladenosine; Ino:, inosine; Ado:, adenosine; 6mA:, N^6^-methyladenine; Hx:, hypoxanthine. (**G**) The predicted binding modes of N^6^-mAdo to the three proteins of interest: YerA (cyan), Bh0637 (violet), and Atu4426 (orange). All the key residues and N^6^-mAdo were presented in sticks form and labeled. The possible hydrogen bonds were shown by the red dashed lines, and the cut-off distance was set to 3.5 Å except for the possible long-hydrogen bond with the main chain Phe117 (with the distance labeled). Abbreviation: MAPDA, N^6^-methyladenosine monophosphate.

To elucidate the structural basis underlying substrate specificity, we conducted docking studies on the predicted YerA/AF-FL-2Zn structure with two potential substrates: N^6^-mAdo and 6mA. Among the multiple docking models generated using Schrödinger’s Glide for N^6^-mAdo, three exhibited high consistency. The sole structural variations were observed in the ribosyl moiety or phosphate group (Figure 4A). The substrate binding pose interacted exclusively with Zn1, situated in close proximity to the general base Asp283 (Figure 4B). These models also displayed similar orientations and were adjacent to the ligand-binding site of the MAPDA/D295N-N^6^-mAMP model (PDB 6IJN). The ligand snugly fitted into the pocket (Figure 4C). Notably, the two TIM-barrel-containing enzymes, MAPDA and YerA, displayed structural similarities, particularly at the substrate-binding sites. MAPDA catalyzes the conversion of N^6^-mAMP to IMP and relies on a single zinc metal; the former substrate differs only by an extra phosphate group. We investigated the possibility of YerA deaminating N^6^-mAMP but failed to detect the product (data not shown). This failure is probably attributed to steric hindrance between YerA’s Phe91 or Leu116 residues and the phosphate, thereby preventing N^6^-mAMP binding (Figure 4A and C).

In stark contrast with the N^6^-mAdo scenario, the predicted binding modes for 6mA were highly diverse and inconsistent. Figure 4D illustrates several possible conformations of this ligand, all significantly differing from the MAPDA complex structure. This variability is likely due to the ligand’s small size, which does not provide sufficient contacts to stabilize it within the binding pocket. Results of the thermal shift analysis indicated that the addition of N^6^-mAdo increased the T_m_ value of apo-YerA by 3°C, whereas other ligands had a minimal impact on the T_m_ (Figure 4E,F). Collectively, these data suggest that the larger ligand N^6^-mAdo serves as a superior substrate for YerA compared with the methylated base form, supported by our deamination activity assays (Figure 3).

Next, we analyzed the substrate recognition mechanism of YerA. As shown in Figure 4B and G, substrate recognition is facilitated by hydrogen bonds from the side chains of Arg252, His89, Gln150, Asp283, and His554 and the main chain of Phe117. Additional hydrophobic contacts are provided by Phe540 and Leu555, while His89 and His554 form partial stacking interactions. These residues are conserved in Bh0637, enhancing the model’s reliability. In contrast, the distant homolog Atu4426 only preserves hydrogen bonds on N6, N7, and N9 of the adenine ring via the conservation of Arg260, Asp289, and His94, enabling precise adenine base recognition. However, residues responsible for hydrophobic interactions are mostly replaced or lost. For instance, Gly569 in Atu224 replaces Phe549 in YerA and poorly superimposes with the latter. The extra contacts on the ribose ring, conferring sugar specificity, are lost or impaired.

Previously, Jiang et al. also investigated the potential binding mode of N^6^-mAdo to YerA [21]. Their model proposed that Asp283, Gly284, and Ser255 might form hydrogen bonds with the substrate’s ribose ring, while Leu116, Phe117, Gln150, Thr181, Thr466, Ser552, His554, and Leu555 contribute to a larger volume of the pocket and a higher hydrophobicity. Although their model differs slightly from ours in terms of key residue positions and potential catalytic roles, the involved residues are consistent. Both models suggest that Phe91 and Gln150 are crucial for deamination reactions, confirmed by subsequent activity tests [21]. Discrepancies may arise from the modeling algorithms: Jiang et al. used I-TASSER for protein structure generation and Glide for complex model generation, whereas we employed the latest protein prediction tool, Alphafold 3, for YerA structure generation and Glide for complex model generation. Furthermore, Jiang et al. found that YerA prefers adenosine over N^6^-mAdo. This aligns with our model, where the N^6^-methyl group barely interacts with the enzyme; the nearest hydrophobic residues, Ile256 and Phe549, are approximately 5.0 Å away.

### The possible role of YerA^Gly380-Arg580^ in catalysis

**Figure 5:**
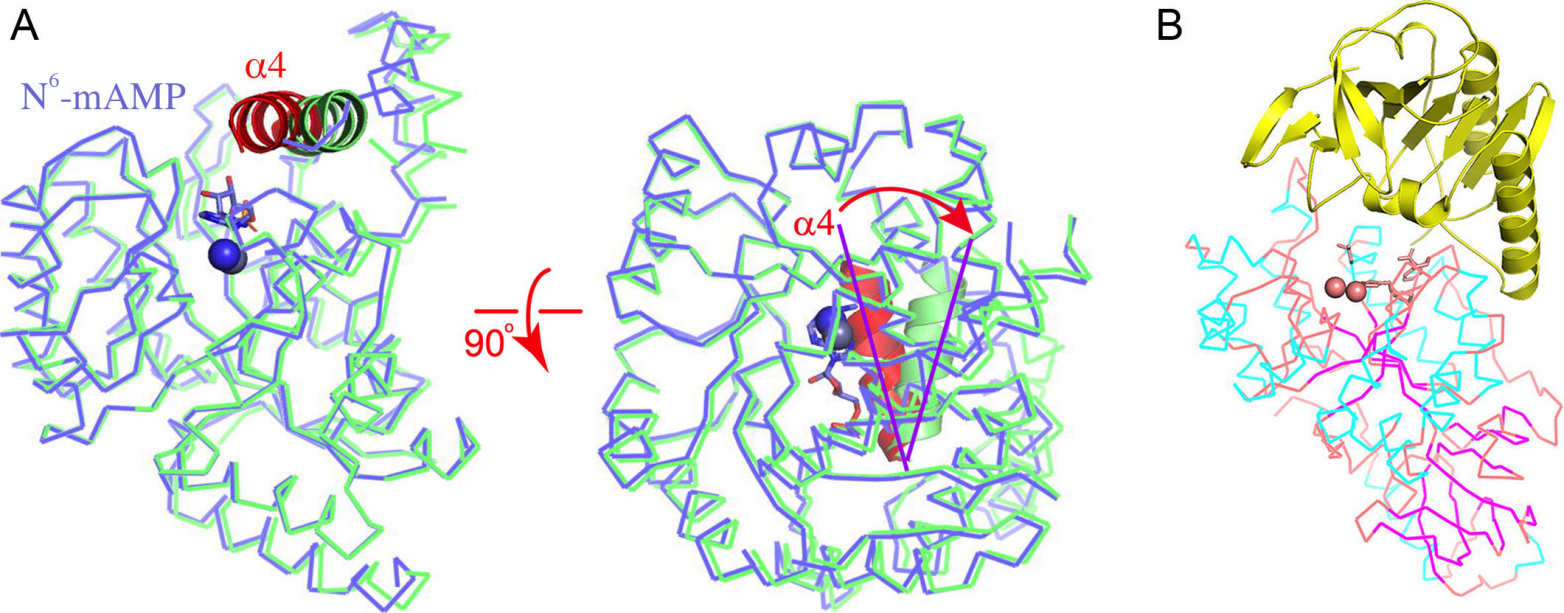
The implications of the C-terminal region of YerA. (**A**) The conformational changes observed in the deamination reaction of MAPDA. The α4-helices in two states of MAPDA are shown in ribbons and colored in red (PDB 6IJN) and green (PDB 6IJM), respectively. The rest of the enzyme is shown in Cα traces. The red arrow indicates the rotation of the helix. (**B**) The YerA structure in Cα trace, from the same view angle as the left panel in (**A**). The yellow ribbon structure represents the C-terminal region. Abbreviation: MAPDA, N^6^-methyladenosine monophosphate.

Regarding the possible role of YerA’s Gly380-Arg580 domain in catalysis, it is intriguing that YerA, as a deaminase acting on a smaller substrate than MAPDA, possesses a large extra C-terminal appendix domain. Attempts to express various YerA constructs without this domain invariably rendered the remaining enzyme insoluble, suggesting a structural role. During the structural comparison of YerA’s Gly380-Arg580 domain with MAPDA, we found that this domain may function as a lid covering YerA’s active site, presumably playing an equivalent role to MAPDA’s α4-helix. This helix undergoes significant conformational changes during MAPDA catalysis. Upon ligand binding, it collapses onto the substrate-binding pocket, sequestering the active site (Figure 5A) [22]. Similar movement has been observed in the enzymatic reaction of guanosine deaminase(GSDA), a cytidine deaminase(CDA)-type deaminase converting guanosine to xanthosine. During deamination, the C-terminal tail of one subunit of the dimeric enzyme inserts into the active site of the other, sealing the active site [25]. Therefore, a bulk water-excluded active site may be crucial for deamination. Considering the independence and relative position of YerA’s Gly380-Arg580 domain to the active site, it may serve as a convenient lid switching between ‘open’ and ‘closed’ conformations (Figure 5B). Additionally, this non-catalytic domain appears unique due to limited homologous structures in the Protein Data Bank (PDB). Further investigation into the evolutionary origin of this domain is warranted.

## Discussion

The bacterial R–M system protects endogenous DNA from degradation by selectively recognizing and cleaving foreign DNA, thereby preventing viral invasion and regulating bacterial growth and death. In the present study, we conducted crystallographic, biochemical, and docking analyses on the deaminase YerA, providing insights into its biochemical characteristics and evolutionary pathways. Our research also investigated the substrate preferences of the enzyme and explored potential ligand-binding modes. Notably, our findings revealed that YerA exhibits a preference for N^6^-mAdo over 6mA, despite its substrate promiscuity.

Given the complexities associated with deaminases, our understanding of these fascinating enzymes remains limited. Current challenges and issues that need to be addressed in the field of deaminases encompass the following two aspects:

Structure–activity relationships: Deaminases exist in various forms, particularly in terms of their tertiary and quaternary structures (e.g. AHS vs. TIM-barrel, monomeric vs. dimeric). Additionally, many deaminases differ in the types and numbers of metals they utilize. For instance, the biological metals of YerA are yet to be determined. Furthermore, the catalytic mechanisms of numerous deaminases remain unclear; both single-metal and binuclear metal catalysis have been proposed, but the underlying reasons remain obscure. Finally, deaminases display distinct sizes and work on different substrates (bases, nucleosides, nucleotides, and nucleic acids) to catalyze various deamination reactions (A-to-I, C-to-U, and G-to-X deamination). Therefore, the evolutionary origins and pathways of such diverse deaminases are intriguing and warrant further investigation.

True biological functions and significance: Nucleic acid deaminases have been extensively studied. For example, adenosine deaminase acting on transfer RNA(ADAT) catalyzes the deamination of tRNA, while adenosine deaminases acting on RNA(ADAR) and apolipoprotein B mRNA editing catalytic polypeptide-like(APOBEC) target ssDNA or dsRNA. In contrast, except for a few cases, the *in vivo* functions of most nucleoside deaminases remain unclear. Notable studies by Chen et al. and Jiang et al. have shed light on the *in vivo* functions of MAPDA and YerA [21,29]. Interestingly, many of these enzymes are specific to certain kingdoms or localize in specific organelles, suggesting their *in vivo* roles. However, a significant challenge is that many knockout experiments have not yielded obvious phenotypes (e.g. MAPDA), making it difficult to ascertain their exact functions.

A multidisciplinary approach involving bioinformatics, biochemistry, structural information, and cell-based assays is essential for comprehensively understanding and utilizing these interesting enzymes. In recent years, base editing techniques utilizing deaminase functions have undergone rapid development. These techniques are advantageous due to their high editing efficiencies, lower off-target effects, and simplicity in implementation, enabling specific C-to-T or A-to-G transitions in genomic DNA across various organisms with single-nucleotide precision [30]. The adenine base editor employs a version of the *E. coli* TadA deaminase generated through direct evolution. YerA has been shown to deaminate both unmethylated and methylated adenosine forms, expanding the functionality of deaminases. However, a challenge with YerA is that it operates at the nucleic acid level. Therefore, enzyme engineering is necessary to enable its binding to DNA, a strategy that has proven successful for many BE deaminases (e.g. TadA). More recently, AI-based protein structure prediction and clustering have facilitated the design of a suite of deaminases for various new nucleic acid substrates, further expanding the deaminase family and the repertoire of possible gene-editing tools [31]. Overall, our study not only contributes to the understanding of the purine metabolic pathway but also holds promise for developing YerA as a gene-editing tool.

## Materials and methods

### Cloning, protein expression, and purification of YerA and truncated mutants

The full-length human *yerA* gene (GenBank accession No. AKL83378) or various truncated forms were amplified from *B. subtilis* cDNA and cloned into the expression vector pET-28a(+) (Novagen) using the NheI and XhoI restriction sites. The constructed vectors were transformed into *E. coli* strain BL21 (DE3) cells. The cells were cultured overnight in Luria–Bertani broth containing 50 mg·ml^-1^ kanamycin; 2 L fresh culture medium was then inoculated with 5 mL overnight culture. At an OD_600_ of 0.2, 200 µM 2, 2′-bipyridyl was added to supplement the growth medium. When the OD_600_ absorbance reached 0.6–0.8, 1.0 mM ZnCl_2_ or MnCl_2_ or Fe(NH4)_2_·(SO4)_2_·6H_2_O was added, followed by induction with 0.2 mM isopropyl-β-D-thiogalactopyranoside, and the culture was shaken at 25℃ overnight. The *E. coli* cells were then harvested by centrifugation at 5000 rpm for 15 min and resuspended in pre-chilled nickel–nitrilotriacetic acid (Ni-NTA) buffer A [20 mM Tris-HCl pH 8.0, 250 mM NaCl, 10 mM imidazole, 1 mM β-mercaptoethanol, 1 mM phenylmethylsulfonyl fluoride (PMSF)]. The cells were disrupted by ultrasonication and the supernatant was obtained by centrifugation at 14,000 rpm for 1 h at 4℃. The supernatant was then applied onto Ni-NTA affinity resin (Qiagen), which had previously been equilibrated with Ni-NTA buffer A. The target protein was eluted with Ni-NTA buffer B (20 mM Tris-HCl pH 8.0, 250 mM NaCl, 250 mM imidazole, 1 mM PMSF). The YerA fractions were pooled and dialyzed in a buffer consisting of 20 mM Tris-HCl pH 8.0, 100 mM NaCl, 1 mM DTT. The dialyzed protein was applied onto a HiTrap Q HP column (GE Healthcare) equilibrated with Q HP buffer A (20 mM Tris-HCl pH 8.0, 50 mM NaCl, 1 mM DTT). YerA protein was eluted with Q HP buffer B consisting of 20 mM Tris-HCl pH 8.0, 1 M NaCl, 1 mM DTT. The N-terminally fused His-tag was cleaved off by the PreScission protease (PSP) treatment overnight at a molar ratio of 80:1 (YerA:PSP), while dialyzing against a buffer containing 20 mM Tris-HCl pH 8.0, 150 mM NaCl. The target proteins without the tag were enriched by collecting the unbound fractions from a HiTrap Histrap column (GE Healthcare), which employed the same buffers as those for Ni-NTA affinity chromatography. The final fractions of YerA were concentrated to 10 mg·ml^-1^ using a Millipore centrifugal filter (molecular-weight cutoff: 30 kDa) and stored at −80℃.

### Crystallization and data collection

Initial crystallization screening was set up by a Mosquito crystallization robot (TTP Labtech) using the sitting-drop vapor-diffusion method with the commercial screens Index, Crystal Screen, and Crystal Screen 2 (Hampton Research), as well as a homemade PEG/ammonium sulfate-based screen, at 25℃ in 96-well plates. Crystallization hits were observed in two to three days, and two crystal forms were obtained: cube-shaped crystals and diamond-shaped crystals. The former were found in a condition consisting of 1.5 M (NH_4_)_2_SO_4_, 0.1 M HEPES pH 7.0, while the latter were found in a condition consisting of 10% PEG 3350, 0.1M NaOAc pH 6.0. After optimization, thick rod-shaped crystals were obtained from a condition consisting of 1.5 M KH_2_PO_4_, 0.1 M HEPES pH 7.0. Crystals were soaked in a freshly made cryoprotective solution containing all of the components of the reservoir solution plus 20% (v/v) glycerol. The soaked crystals were mounted on nylon loops and flash-cooled in liquid nitrogen. Using an in-house Oxford Diffraction Xcalibur Nova diffractometer operating at 50 kV and 0.8 mA, a full native dataset (a total of 99 frames) was collected with a rotation of 0.75 per frame at 100 K. The data were recorded with a 165-mm Onyx CCD detector and were processed and scaled using CrysAlis^Pro^ (Oxford Diffraction) and SCALA from the CCP4 suite (Table 1) [32].

### Structure determination

The space group of the crystal was *P*4_1_2_1_2, and the crystal diffracted to 2.1 Å resolution with a completeness of 99.9% (Table 1). The structure was solved by molecular replacement using PHENIX [33] with the coordinates of the C-terminal region of the AlphaFold 3-predicted YerA structure as the search model [34]. The initial model was extended by PHENIX using the autobuild option, and the resulting model was further built manually according to the electron-density map with Coot [35]. Multiple cycles of refinement alternating with model rebuilding were carried out by phenix.refine [33]. The final R factor was 22.6% (R_free_ = 27.1%). The Ramachandran plot of the final model has 95.5%, 4.5%, and 0% of the residues in the most favored, generously allowed and disallowed regions (Table 1). The final model was validated by SFCHECK and PROCHECK [36,37]. All figures were produced with PyMOL (http://www.pymol.org), and the charge distribution on the protein surface was calculated by APBS [38]. The secondary structure of YerA-SF was prepared by ESPript (http://espript.ibcp.fr) [39]. The atomic coordinates and structure factors have been deposited in the PDB (http://www.rcsb.org/) as entry 9JQL.

### Mass spectrometry

Samples were prepared by mixing 10 mM YerA with 5 mM ligands and were incubated for 5 h at 37°C. The samples were diluted 100-fold by ddH_2_O before injection. The reaction products were either detected by an LC Q-TOF (Synapt G2 Si, Waters) or a Tims TOF spectrometer (Bruker) with an electrospray ion source operating in the positive ion mode. The instrument parameters for the mass spectrometer were as follows: ion polarity = positive; scan range for full scan data collection = 100–1500 m/z; set capillary = 4500 V; set end plate offset = −500 V; set collision cell RF = 900 Vpp; set nebulizer = 0.6 Bar; set dry heater = 200°C; set dry gas = 6.0 L/min.

### Molecular docking

We utilized Schrödinger’s Protein Preparation Wizard to optimize the YerA/AF-FL-2Zn structure by adding missing hydrogen atoms, optimizing bond lengths and angles, and assigning partial charges. The structures of the ligand molecules were downloaded for docking from the PubChem database [40]. The 3D structures of potential ligands were created using Schrödinger’s LigPrep module. A grid box encompassing the TIM domain of YerA was defined. Schrödinger’s Glide docking software was employed to identify potential binding orientations and conformations.

### Thermal shift analysis

The 20 μL sample containing 15 μM apo-YerA, 3 mM ligand, and 2 μL of 2 × SYPRO orange fluorescent dye (Sigma-Aldrich) was incubated in a 96-well PCR plate on ice. The plate was incubated in the StepOnePlus Real-Time PCR System (Life Technologies) at 25°C for 10 min and then gradually heated to 95°C at a heating rate of 1 °C·min^-1^. The fluorescence signals of the dyes at 490/530 nm wavelengths (excitation and emission, respectively) during thermal denaturation were recorded every 30 s. The assays were conducted in triplicates for all the mutants and the control.

## Supplementary material

online supplementary figure 1.

## Data Availability

Atomic coordinates and structure factors for the reported crystal structure has been deposited with the Protein Data Bank under accession number 9JQL.

## Abbreviations

ADAR: adenosine deaminases acting on RNA
ADAT: adenosine deaminase acting on transfer RNA
ADE: adenine deaminase
AHS: amidohydrolase superfamily
APOBEC: apolipoprotein B mRNA editing catalytic polypeptide-like
CDA: cytidine deaminase
GSDA: guanosine deaminase
MAPDA: N^6^-methyladenosine monophosphate deaminase
N^6^-mAdo: N^6^-methyladenosine
PDB: Protein Data Bank
RMSD: root-mean-square deviation
TIM: triose-phosphate isomerase
6mA: N^6^-methyladenine
