# Supplementary material for: The C-terminal structure of the N6-methyladenosine deaminase YerA and its role in deamination
Source: Biochem J. 2025 Feb 12;482(4):BCJ20240728. doi: 10.1042/BCJ20240728 (PMC12133300; doi:10.1042/BCJ20240728)
Supplement: online supplementary figure 1. [file bcj-482-4-BCJ20240728-s001.docx]

**Supplementary Information**

**The C-terminal structure of the N^6^-methyladenosine deaminase YerA and its role in deamination**

Qian Jia^1^, Hui Zeng^1^, Nan Xiao^1^, Jing Tang^1^, Shangfang Gao^1^ and Wei Xie^1,^*

^1^ MOE Key Laboratory of Gene Function and Regulation, State Key Laboratory for Biocontrol, School of Life Sciences, Sun Yat-Sen University, Guangzhou, Guangdong, 510006, People's Republic of China

* To whom correspondence should be addressed. Tel: 862039332943; Fax: 862039332847; Email: [xiewei6@mail.sysu.edu.cn](mailto:xiewei6@mail.sysu.edu.cn,)

Table of Contents

Supplementary Figure S1 and Table S1.


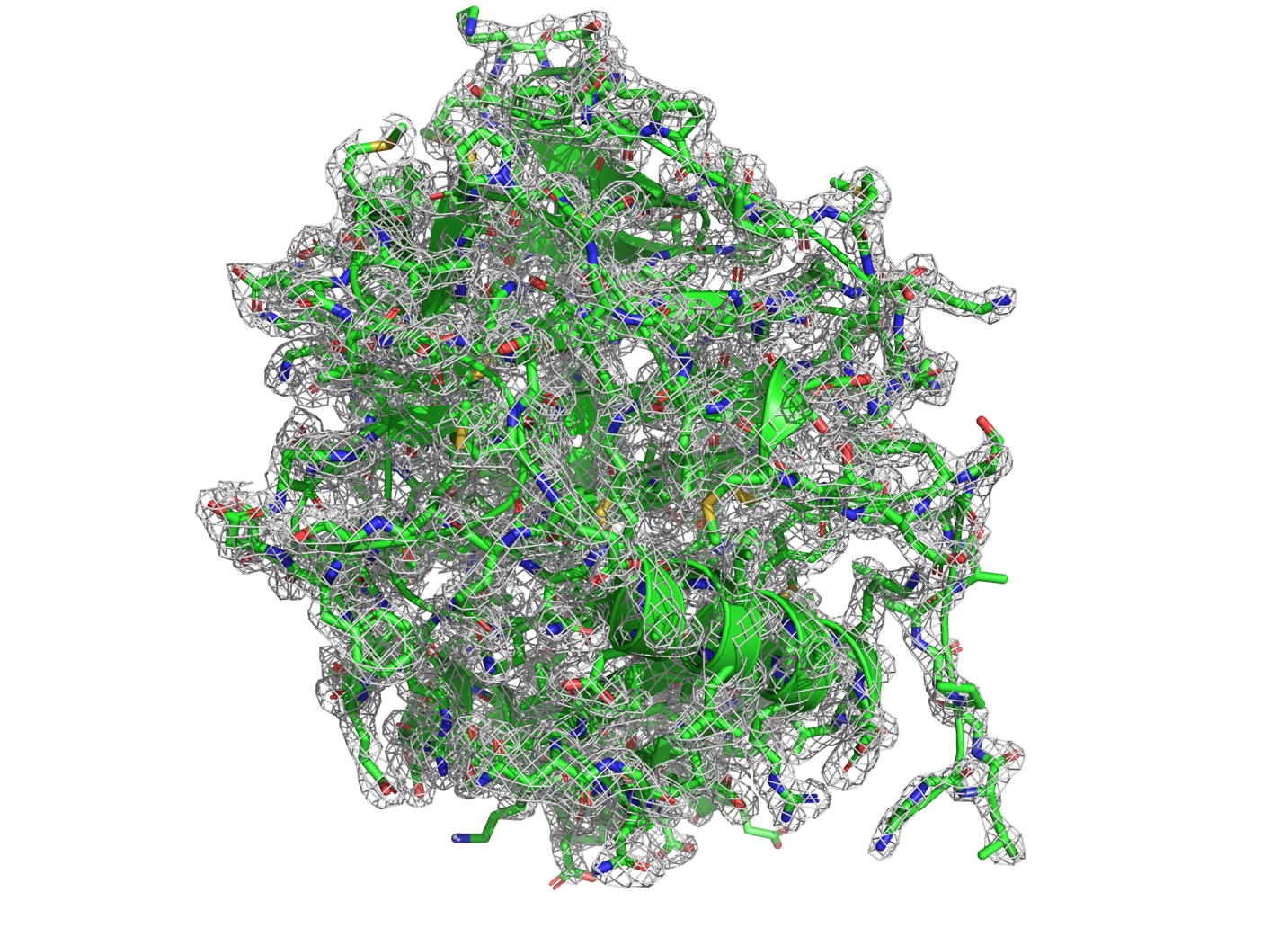


Figure S1. The electron density of YerA^Gly380-Arg580^. The 2Fo-Fc map was countoured at 1 σ.

| **Constructs**  **(pET-28a(+))** | **Sense Primers** | **Antisense Primers** |
| --- | --- | --- |
| FL | GCGGCAGCGCTAGCATGTCCGAACGCACCTTTAACTGGA | TTGCACTTCTCGAGTTAACGCATTATCGATGGAAAGAG |
| Ala15-Gly182 | GCGGCAGCGCTAGCGCACAGGTTGATGTTGTAGACT | TTGCACTTCTCGAGTTATCCGGTCATTTCTCCGCC |
| Ala15-Asp327 | GCGGCAGCGCTAGCGCACAGGTTGATGTTGTAGACT | TTGCACTTCTCGAGTTAATCCATTTGGTAATACTT |
| Ala15-Pro576 | GCGGCAGCGCTAGCGCACAGGTTGATGTTGTAGACT | TTGCACTTCTCGAGTTATGGAAAGAGTACAGTTTTTTTCAT |
| Ala15-Arg580 | GCGGCAGCGCTAGCGCACAGGTTGATGTTGTAGACT | TTGCACTTCTCGAGTTAACGCATTATCGATGGAAAGAG |
| Ala15-Pro384 | GCGGCAGCGCTAGCGCACAGGTTGATGTTGTAGACT | TTGCACTTCTCGAGTTACGGGACAAGACCGCCTTT |
| Pro26-Gln325 | GCGGCAGCGCTAGCCCAACGCTCCTTTTACGGAAT | TTGCACTTCTCGAGTTATTGGTAATACTTTGCGATATTAAA |
| Pro26-Ala371 | GCGGCAGCGCTAGCCCAACGCTCCTTTTACGGAAT | TTGCACTTCTCGAGTTACGCCTTCAAATCACAGCC |
| Pro26-Gly380 | GCGGCAGCGCTAGCCCAACGCTCCTTTTACGGAAT | TTGCACTTCTCGAGTTAGCCTTTGTGCCAGTCCGTCT |
| Pro26-Asp390 | GCGGCAGCGCTAGCCCAACGCTCCTTTTACGGAAT | TTGCACTTCTCGAGTTAATCATATGAAAGCTCAAGCGG |
| Pro26-Arg580 | GCGGCAGCGCTAGCCCAACGCTCCTTTTACGGAAT | TTGCACTTCTCGAGTTAACGCATTATCGATGGAAAGAG |
| Gly76-Gly306 | GCGGCAGCGCTAGCGGAAAATACATTGTGCCA | TTGCACTTCTCGAGGCCTTCTTCTAACGCAATGC |
| Gly76-Gly366 | GCGGCAGCGCTAGCGGAAAATACATTGTGCCA | TTGCACTTCTCGAGTTAGCCGTTTTCACGCAGAAT |
| Gly76-Arg580 | GCGGCAGCGCTAGCGGAAAATACATTGTGCCA | TTGCACTTCTCGAGTTAACGCATTATCGATGGAAAGAG |
| Pro81-Ser456 | GCGGCAGCGCTAGCCCAGGTTATATTGAACCG | TTGCACTTCTCGAGTTAGCTTGAGGCAAACCCTTT |
| Gly82-Ala285 | GCGGCAGCGCTAGCGGTTATATTGAACCGCAC | TTGCACTTCTCGAGTTATGCGCCATCTGTCGTGTA |
| Pro90-Asp328 | GCGGCAGCGCTAGCCCTTTTCAAATATATAAT | TTGCACTTCTCGAGTTAATCATCCATTTGGTAATA |
| Asn95-Thr373 | GCGGCAGCGCTAGCAATCCCCAAACACTGGCG | TTGCACTTCTCGAGTTATGTAAACGCCTTCAAATC |
| Asn95-Gly380 | GCGGCAGCGCTAGCAATCCCCAAACACTGGCG | TTGCACTTCTCGAGTTAGCCTTTGTGCCAGTCCGTCT |
| Thr181-Arg580 | GCGGCAGCGCTAGCACCGGATGGCCTCGCCTG | TTGCACTTCTCGAGTTAACGCATTATCGATGGAAAGAG |
| Ala235-Arg580 | GCGGCAGCGCTAGCACAGGTGATGAAGTGATG | TTGCACTTCTCGAGTTAACGCATTATCGATGGAAAGAG |
| Ser286-Arg580 | GCGGCAGCGCTAGCACCCCGAATTTCTATAAA | TTGCACTTCTCGAGTTAACGCATTATCGATGGAAAGAG |
| Gln325-Arg580 | GCGGCAGCGCTAGCCAAATGGATGATTATTTAGGAGT | TTGCACTTCTCGAGTTAACGCATTATCGATGGAAAGAG |
| Gly366-Arg580 | GCGGCAGCGCTAGCGGCTGTGATTTGAAGGCGTTT | TTGCACTTCTCGAGTTAACGCATTATCGATGGAAAGAG |
| Gly380-Arg580 | GCGGCAGCGCTAGCGGCGGTCTTGTCCCGC | TTGCACTTCTCGAGTTAACGCATTATCGATGGAAAGAG |

Table S1. Primers used in constructs.
